# A Simple and Efficient Two-Dimensional High-Speed Counter-Current Chromatography Linear Gradient and Isocratic Elution Modes for the Preparative Separation of Coumarins from Roots of *Toddalia asiatica* (Linn.) Lam.

**DOI:** 10.3390/molecules26195986

**Published:** 2021-10-02

**Authors:** Wenya Ma, Iftikhar Ali, Yali Li, Hidayat Hussain, Huanzhu Zhao, Xuan Sun, Lei Xie, Li Cui, Daijie Wang

**Affiliations:** 1 College of Life Science, Shandong Normal University, Jinan 250014, China; wenyama97@126.com; 2 School of Pharmaceutical Sciences and Key Laboratory for Applied Technology of Sophisticated Analytical Instruments of Shandong Province, Shandong Analysis and Test Center, Qilu University of Technology (Shandong Academy of Sciences), Jinan 250014, China; iftikhar.ali@kiu.edu.pk (I.A.); 17864170352@163.com (H.Z.); sx951009@163.com (X.S.); xieleito@gmail.com (L.X.); 3 Department of Chemistry, Karakoram International University, Gilgit 15100, Pakistan; 4 Key Laboratory of the Innovative, Development of Functional Staple and the Nutritional, Intervention for Chronic Disease, China National Research Institute of Food & Fermentation Industries Co., Ltd., Beijing 100015, China; cnliyali@163.com; 5 Department of Bioorganic Chemistry, Leibniz Institute of Plant Biochemistry, Weinberg 3, D-06120 Halle (Saale), Germany; hussainchem3@gmail.com

**Keywords:** *Toddalia asiatica* (L.) Lam., high-speed counter-current chromatography, coumarins, linear gradient

## Abstract

*Toddalia asiatica* (L.) Lam. (Rutaceae) has shown a broad spectrum of biological properties, such as anti-inflammatory, antioxidant, antimicrobial, anti-HIV, and anticancer properties. The present study is concerned with the separation of the main components with broad partition coefficients (*K*_D_ values) from *T. asiatica*, using linear gradient high-speed counter-current chromatography (LGCCC) combined with an off-line two-dimensional (2D) mode. Similar to the binary gradient HPLC, the LGCCC mode is operated by the adjustment of the proportion between the mobile phase of 5:5:1:9 (*v*/*v*) (pump A) and 5:5:4.5:5.5 (*v*/*v*) (pump B) in an *n*-hexane/ethyl acetate/methanol/water solvent system. The off-line 2D-CCC mode was used in this study for the secondary separation of two similar *K*_D_ value compounds with *n*-hexane/ethyl acetate/methanol/water (5:5:4:6, *v*/*v*). Notably, six coumarins, namely, tomentin (**1**), toddalolactone (**2**), 5,7,8-trimethoxycoumarin (**3**), mexoticin (**4**), isopimpinellin (**5**), and toddanone (**6**), were efficiently separated. The structures of the pure compounds were elucidated by spectral techniques and compared with the literature.

## 1. Introduction

*Toddalia asiatica* (L.) Lam. (Rutaceae) is used in traditional medicine and is reported to treat wounds, pain, swelling, fever, malaria, diarrhea, cancer, coughs, and stomach infections. In addition, this plant exhibits anti-inflammatory, antioxidant, antibacterial, and antifungal properties [1,2,3,4]. Furthermore, *T. asiatica* is reported to treat epilepsy [5]. The literature reveals that various natural products, including alkaloids, quinolone derivatives, coumarins, and essential oils, are derived from *T. asiatica*, which has shown antidiabetic [6], anti-HIV [7], anticancer [8], antibacterial [9], antiplasmodial [10], antifungal, antibacterial [4], anti-inflammatory [11], and antiviral [12] properties.

Previous studies show that different methods, such as GC/MS [3,13,14,15], HPLC [16,17,18], UPLC-MS/MS [19] UPLC-QTOF-MS/MS [17], and HPTLC [20], can be used for the purification of the chemical constitutes in *T. asiatica*. High-speed counter-current chromatography (HSCCC) using the *n*-hexane/ethyl acetate/methanol/water (10:10:11:9, *v*/*v*) solvent system is reported for the separation of isopimpinellin, pimpinellin, and phellopterin from *T. asiatica* [21]. Recently, in a previous study conducted by our group, the *n*-hexane/ethyl acetate/methanol/water (5:5:3:7, *v*/*v*) biphasic solvent system was successfully used to separate toddalolactone, leptodactylone, haplopine, skimmianine, and 5-methoxydictamnine from *T. asiatica* [22].

**Figure 1:**
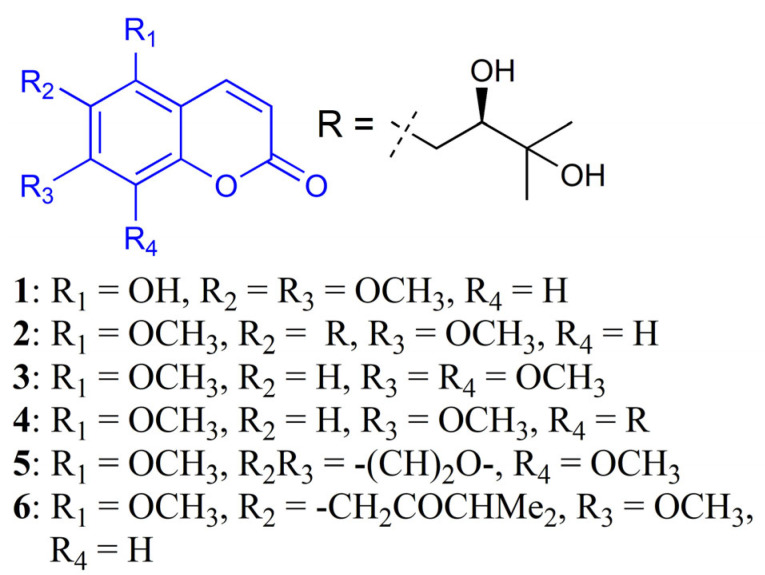
Chemical structures of compounds **1**–**6** separated from *T. asiatica.*

In this study, a more efficient separation of components from *T. asiatica* is established using a two-dimensional high-speed counter-current chromatography (HSCCC) linear gradient and isocratic elution modes. Similar to the binary gradient HPLC, the LGCCC mode is operated by an adjustment of the proportion between the mobile phase of 5:5:1:9 (*v*/*v*) (pump A) and 5:5:4.5:5.5 (*v*/*v*) (pump B) in an *n*-hexane/ethyl acetate/methanol/water solvent system. The off-line 2D-CCC mode was used for the secondary separation of two similar *K*_D_ value compounds with *n*-hexane/ethyl acetate/methanol/water (5:5:4:6, *v*/*v*). Notably, six coumarins, namely, tomentin (**1**), toddalolactone (**2**), 5,7,8-trimethoxycoumarin (**3**), mexoticin (**4**), isopimpinellin (**5**), and toddanone (**6**), were efficiently separated (Figure 1). Recently, HSCCC has been used to effectively separate a variety of components from plant species [23,24,25].

The structures of the pure compounds were elucidated by ^1^H- and ^13^C-NMR, and ESI-MS (positive/negative ion mode). Furthermore, the spectral information was compared with that of previous studies.

## 2. Results

### 2.1. Selection of the HSCCC Solvent System

A successful separation of the target compounds using HSCCC requires a careful search for a suitable two-phase solvent system to provide an ideal range of *K*_D_ values from 0.5 to 2. Higher *K*_D_ values may lead to extended elution times and excessively broad peaks, whereas lower *K*_D_ values might lead to poor peak resolution [26]. In the present experiment, a series of solvent systems were tested with *n*-hexane/ethyl acetate/methanol/water (5:5:X:10-X, *v*/*v*). As shown in Table 1, the *K*_D_ values of the six target compounds were successfully spread.

When the solvent system *n*-hexane/ethyl acetate/methanol/water (5:5:1:9, *v*/*v*) was used, the *K*_D_ values of compounds **1** and **2** were suitable, whereas compounds **3–6** were larger than 5 and needed a long elution time. On the other hand, when the ratio of methanol was increased, the *K*_D_ values decreased gradually. When the solvent system *n*-hexane/ethyl acetate/methanol/water (5:5:4:6, *v*/*v*) was used, the *K*_D_ values of compounds **3** and **4** were suitable, whereas compounds **1** and **2** were smaller than 0.5 and had a poor resolution. Furthermore, compounds **5** and **6** were slightly larger with a long elution time. When the solvent system *n*-hexane/ethyl acetate/methanol/water (5:5:5:5, *v*/*v*) was used, the *K*_D_ values of compounds **5** and **6** were suitable, whereas compounds **1–4** were smaller. The above results indicated that the *K*_D_ values spanned a wide range, and so it was difficult to separate them with a single solvent system. LGCCC separation was chosen to separate coumarins from the roots of *T. asiatica*. According to the results of the tested *K*_D_ values, the lower phase of *n*-hexane/ethyl acetate/methanol/water (5:5:1:9, *v*/*v*) was used as mobile phase A, while *n*-hexane/ethyl acetate/methanol/water (5:5:5:5, 5:5:6:4, *v*/*v*) was selected as mobile phase B to build the 5:5:X:10-X (*v*/*v*) solvent system for LGCCC elution.

### 2.2. Optimization of HSCCC Conditions

**Figure 2:**
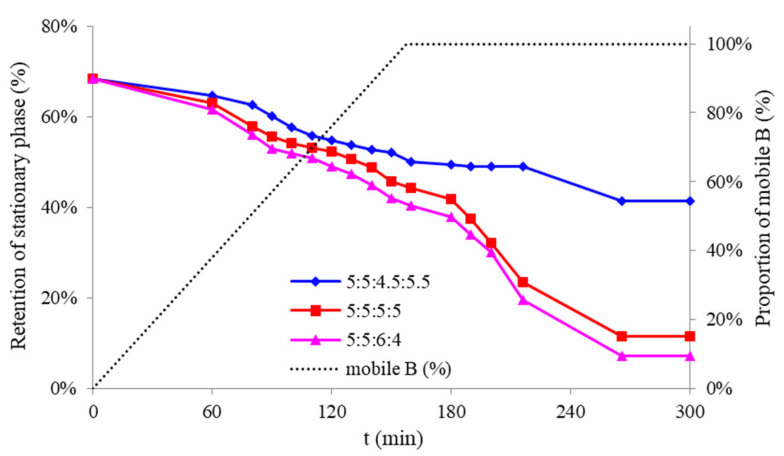
The linear gradient CCC procedure tested with different solvent systems. Stationary phase: upper phase of *n*-hexane/ethyl acetate/methanol/water (5:5:1:9, *v*/*v*); mobile phase: lower phase of *n*-hexane/ethyl acetate/methanol/water (5:5:1:9, *v*/*v*) as mobile A and lower phase of *n*-hexane/ethyl acetate/methanol/water (5:5:4.5:5.5, 5:5:5:5, 5:5:6:4, *v*/*v*) as mobile B; linear elution condition: 0–157.5 min, 0–100% B, and 157.5–300 min, 100% B; and flowrate: 5.0 mL/min.

Figure 2 shows the retention of stationary phase (*R*_S_) testing of the LGCCC mode. The stationary phase was used as the upper phase of *n*-hexane/ethyl acetate/methanol/water (5:5:1:9, *v*/*v*), while the mobile phase was set as the mobile phase of *n*-hexane/ethyl acetate/methanol/water (5:5:1:9, *v*/*v*) (mobile phase A). Another LGCCC mobile phase (mobile phase B) was tested with different *n*-hexane/ethyl acetate/methanol/water solvent systems that had a linear elution condition of 0–157.5 min, 0–100% B, and 157.5–300 min, 100% B. The CCC flow direction was in head-to-tail mode with a flowrate of 5.0 mL/min. When mobile phase B was used as *n*-hexane/ethyl acetate/methanol/water (5:5:6:4, *v*/*v*), the loss of *R*_S_ was obvious with only 7.14% at 300 min. This indicated that it would affect the separation effect with the loss of the stationary phase. When the *n*-hexane/ethyl acetate/methanol/water (5:5:5:5, *v*/*v*) solvent system was used, the loss of *R*_S_ was improved; however, the *R*_S_ was only 11.43%. Although the *n*-hexane/ethyl acetate/methanol/water (5:5:5:5, *v*/*v*) solvent system was suitable for separation compounds **5** and **6**, the loss of *R*_S_ indicated that it was not suitable to build an LGCCC solvent system. In this case, mobile phase B was tested as *n*-hexane/ethyl acetate/methanol/water (5:5:4.5:5.5, *v*/*v*) and a good *R*_S_ result of over 40% was obtained at 300 min. When *n*-hexane/ethyl acetate/methanol/water (5:5:4:6, *v*/*v*) and other hydrophilic mobile phases were used, the elution time was longer with an increased amount of solvent consumption. The above results indicate that a careful evaluation of the *R*_S_ value of the two mobile phases for a suitable LGCCC separation is necessary.

**Figure 3:**
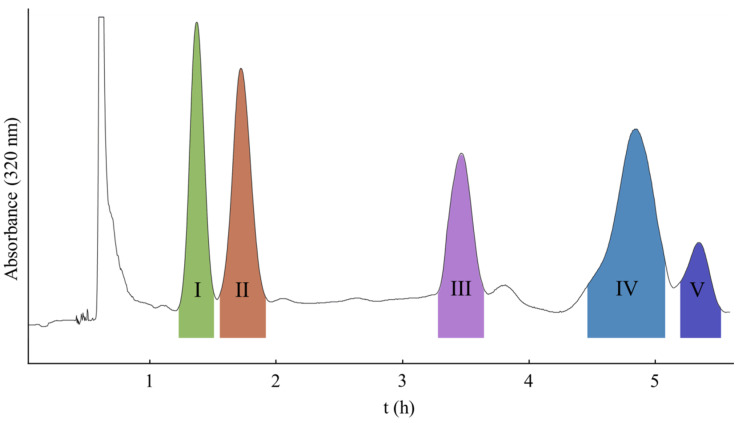
The linear gradient CCC procedure on the separation of coumarins from the roots of *T. asiatica*. Stationary phase: upper phase of *n*-hexane/ethyl acetate/methanol/water (5:5:1:9, *v*/*v*); mobile phase: lower phase of *n*-hexane/ethyl acetate/methanol/water (5:5:1:9, *v*/*v*) as mobile A and lower phase of *n*-hexane/ethyl acetate/methanol/water (5:5:4.5:5.5, *v*/*v*) as mobile B; linear elution condition: 0–157.5 min, 0–100% B, and 157.5–360 min, 100% B; flowrate: 5.0 mL/min; and detection wavelength: 320 nm.

Figure 3 shows the HSCCC of the crude extract of coumarins from the roots of *T. asiatica*. The LGCCC mobile phase comprised of mobile phase A and B with a linear gradient elution of 0–157.5 min, 0–100% B, and 157.5–360 min, 100% B. The upper phase of *n*-hexane/ethyl acetate/methanol/water (5:5:1:9, *v*/*v*) was used as the stationary phase in head-to-tail elution mode with a flowrate 5.0 mL/min. The retention of the stationary phase was 40.3% with a total separation time of 360 min. As shown in Figure 3, after one-step separation, five main peaks were obtained: peak I (19.3 mg), peak II (31.4 mg), peak III (29.9 mg), peak IV (36.3 mg), and peak V (8.7 mg). Peak I, peak II, peak IV, and peak V correspond to compounds **2**, **1**, **5,** and **6,** respectively, each with the purities over 98%, as determined by HPLC. Peak III was a mixture of compounds **3** and **4**.

**Figure 4:**
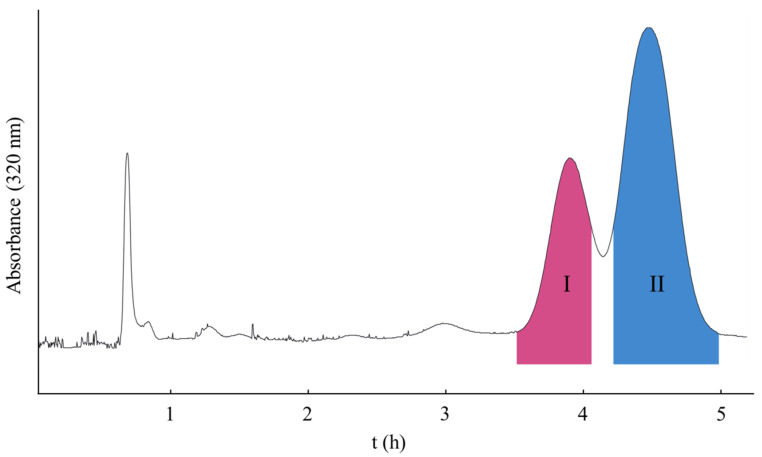
The off-line dimensional CCC procedure on the separation of peak III in Figure 2; solvent system: *n*-hexane/ethyl acetate/methanol/water (5:5:4:6, *v*/*v*); flowrate: 2.0 mL/min; and detection wavelength: 320 nm.

**Figure 5:**
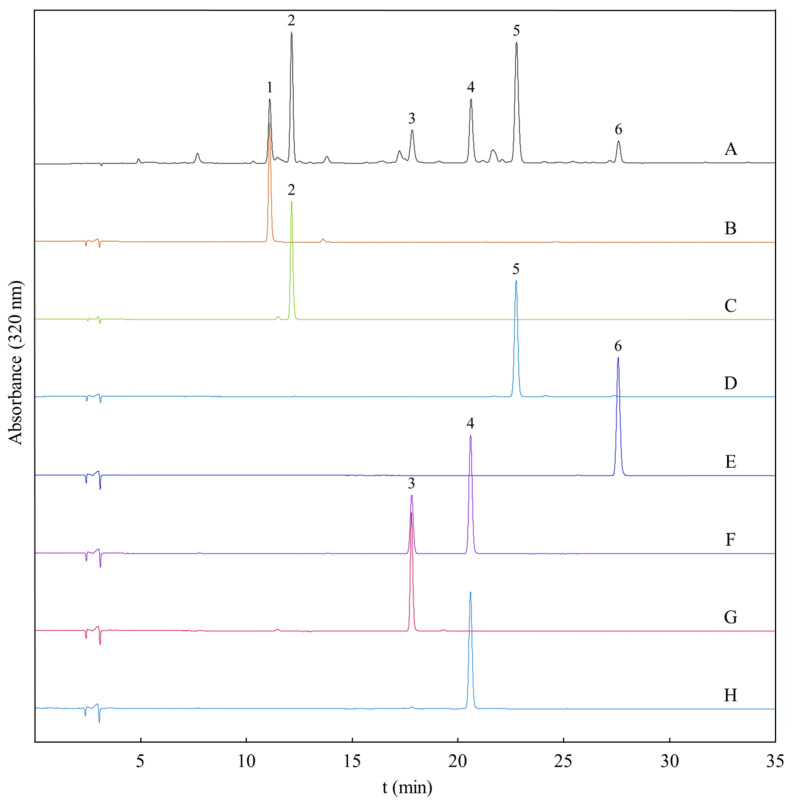
HPLC chromatograms of the crude extract and the isolated coumarins. Experimental conditions: Zobax SB C_18_ column (5 μm, 4.6 mm × 250 mm, i.d.); mobile phase: water (**A**) and acetonitrile (**B**) in a linear elution mode of 0–30 min, 22–55% B; 30–31 min, 55–95% B; and 34–35 min, 95–22% B. Flowrate: 1.0 mL/min; and detection: 320 nm. (**A**): crude extract; (**B**): peak II in Figure 3; (**C**): peak I in Figure 3; (**D**): peak IV in Figure 3; (**E**): peak V in Figure 3; (**F**): peak III in Figure 3; (**G**): peak I in Figure 4; and (**H**): peak II in Figure 4.

Figure 4 shows the off-line dimensional separation of peak III in Figure 3, in which a traditional HSCCC mode with a solvent system of *n*-hexane/ethyl acetate/methanol/water (5:5:4:6, *v*/*v*) was used in head-to-tail elution mode with a flowrate of 2.0 mL/min. The upper phase was used as the stationary phase. The retention of the stationary phase was 61.3% with a total separation time of 300 min. As shown in Figure 4, two peaks were obtained with I (7.9 mg) and II (16.7 mg) and corresponded to compounds **3** and **4**. The purities were over 98%, as determined by HPLC (Figure 5).

### 2.3. Structure Identification

Tomentin **1** [26], (peak 1 in Figure 5), C_11_H_10_O_5_, ESI-MS (positive ion mode) *m*/*z* 223.0657 [M + H]^+^. ^1^H-NMR (DMSO-*d*_6_, 400 MHz) *δ*_H_: 8.83 (1H, br s, OH-5), 8.00 (1H, d, *J* = 9.7 Hz, H-4), 6.67 (1H, s, H-8), 6.15 (1H, d, *J* = 9.7, H-3), 3.92 (3H, s, -OCH_3_), 3.89 (3H, s, -OCH_3_). ^13^C-NMR (DMSO-*d*_6_, 100 MHz) *δ*_C_: 160.6 (C-2), 152.3 (C-7), 149.2 (C-9), 143.9 (C-5), 139.5 (C-4), 127.6 (C-6), 110.9 (C-3), 103.4 (C-10), 93.2 (C-8), 56.9 (-OCH_3_), 56.8 (-OCH_3_).

Toddalolactone **2 [27,28]**, (peak 2 in Figure 5), C_16_H_20_O_6_, ESI-MS (positive ion mode) *m*/*z* 309.1328 [M + H]^+^. ^1^H-NMR (DMSO-*d*_6_, 400 MHz) *δ*_H_: 8.01 (1H, d, *J* = 9.6 Hz, H-4), 6.85 (1H, s, H-8), 6.28 (1H, d, *J* = 9.6 Hz, H-3), 3.87 (3H, s, -OMe), 3.85 (3H, s, -OMe), 3.55 (1H, merged with solvent peak, H-2′), 3.17 (1H, H-1′a), 2.66 (1H, m, H-1′b), 2.52 (1H, br s, -OH), 1.81 (1H, br s, -OH), 1.13 (6H, merged s, -CH_3_). ^13^C-NMR (DMSO-*d*_6_, 100 MHz) *δ*_C_: 162.0 (C-2), 160.4 (C-7), 156.0 (C-5), 154.3 (C-9), 139.8 (C-4), 119.2 (C-6), 111.8 (C-3), 106.8 (C-10), 95.4 (C-8), 75.8 (C-2′), 72.1 (C-3′), 63.1 (7-OMe), 56.4 (5-OMe), 25.9 (C-5′), 25.6 (C-4′), 25.4 (C-1′).

5,7,8-Trimethoxycoumarin **3 [29]**, (peak 3 in Figure 5), C_12_H_12_O_5_, ESI-MS (positive ion mode) *m*/*z* 237.0727 [M + H]^+^. ^1^H-NMR (DMSO-*d*_6_, 400 MHz) *δ*_H_: 8.02 (1H, d, *J* = 9.7 Hz, H-4), 6.69 (1H, s, H-6), 6.19 (1H, d, *J* = 9.7 Hz, H-3), 3.95 (3H, s, -OCH_3_, H-8), 3.93 (3H, s, -OCH_3_, H-5), 3.74 (3H, s, -OCH_3_, H-7). ^13^C-NMR (DMSO-*d*_6_, 100 MHz) *δ*_C_: 160.9 (C-2), 156.6 (C-5), 152.6 (C-7), 148.3 (C-4), 139.5 (C-8b), 129.7 (C-8), 111.2 (C-3), 103.4 (C-8a), 93.2 (C-6), 61.2 (-OCH_3_), 57.0 (-OCH_3_), 56.9 (-OCH_3_).

Mexoticin **4 [30]**, (peak 4 in Figure 5), C_16_H_20_O_6_, ESI-MS (positive ion mode) *m*/*z* 309.1285 [M + H]^+^. ^1^H-NMR (DMSO-*d*_6_, 400 MHz) *δ*_H_: 8.00 (1H, d, *J* = 9.6 Hz, H-4), 6.87 (1H, s, H-6), 6.27 (1H, d, *J* = 9.6 Hz, H-3), 3.87 (3H, s, OCH_3_), 3.85 (3H, s, OCH_3_), 3.72 (1H, m, H-2′), 2.72 (1H, dd, 13.3, 10.1 Hz, H-1′a), 2.67 (1H, dd, *J* = 13.3, 2.6 Hz, H-1′b), 1.15 (3H, s, CH_3_, H-3′), 1.13 (3H, s, CH_3_, H-3′). ^13^C-NMR (DMSO-*d*_6_, 100 MHz) *δ*_C_: 162.3 (C-2), 160.6 (C-7), 156.4 (C-5), 154.6 (C-9), 140.1 (C-4), 119.4 (C-10), 112.2 (C-3), 107.0 (C-8), 95.8 (C-6), 77.5 (C-2′), 73.3 (C-3′), 63.5 (OCH_3_, C-5), 56.8 (OCH_3_, C-7), 25.9 (C-1′), 22.1 (C-5′), 20.6 (C-4′).

Isopimpinellin **5 [31]**, (peak 5 in Figure 5), C_12_H_10_O_2_, ESI-MS (positive ion mode) *m*/*z* 247.0600 [M + H]^+^. ^1^H-NMR (DMSO-*d*_6_, 400 MHz) *δ*_H_: 8.19 (1H, d, *J* = 9.8 Hz, H-4), 8.10 (1H, d, *J* = 2.1 Hz, H-2′), 7.40 (1H, d, *J* = 2.1 Hz. H-3′), 6.35 (1H, d, *J* = 9.8 Hz, H-3), 4.18 (3H, s, OCH_3_), 4.04 (3H, s, OCH_3_). ^13^C-NMR (DMSO-*d*_6_, 100 MHz) *δ*_C_: 159.5 (C-2), 149.4 (C-7), 146.3 (C-2′), 144.3 (C-5), 143.0 (C-8a), 139.7 (C-4), 127.1 (C-8), 114.4 (C-6), 112.5 (C-3), 106.8 (C-4a), 105.6 (C-3′), 61.2 (OCH_3_), 60.8 (OCH_3_).

Toddanone **6 [32]**, (peak 6 in Figure 4), C_16_H_18_O_5_, ESI-MS (positive ion mode) *m*/*z* 291.1184 [M + H]^+^. ^1^H-NMR (DMSO-*d*_6_, 400 MHz) *δ*_H_: 8.01 (1H, d, *J* = 9.7 Hz, H-4), 6.90 (1H, s, H-8), 6.30 (1H, d, *J* = 9.7 Hz, H-3), 3.82 (3H, s, OCH_3_, H-7), 3.80 (2H, s, H-11), 3.73 (3H, s, OCH_3_, H-5), 2.79 (1H, m, H-13), 1.09 (6H, d, *J* = 6.90 Hz, 2CH_3_, H-14, H-15). ^13^C-NMR (DMSO-*d*_6_, 100 MHz) *δ*_C_: 211.4 (C-12), 161.5 (C-7), 160.5 (C-2), 156.2 (C-5), 155.3 (C-9), 139.7 (C-4), 115.0 (C-6), 112.6 (C-3), 106.9 (C-10), 96.0 (C-8), 63.6 (OCH_3_, C-5), 56.9 (OCH_3_, C-7), 40.3 (C-13), 35.6 (C-11), 18.6 (C-14), 18.6 (C-15).

## 3. Materials and Methods

### 3.1. Reagents and Materials

HPLC-grade acetonitrile was obtained from the Tedia Company, Inc. (Fairfield, CT, USA). The solvents used for extraction and separation were of analytical grade (Sinopharm Chemical Reagent Co., Ltd., Shanghai, China). The deionized water was disposed by an osmosis Milli-Q system.

The roots of *T. asiatica* were obtained from a local medicine store in Zhaoqing (Guangxi, China) and identified by Dr. Xiao Wang (Shandong Analysis and Test Center) with a voucher specimen (2018060911) in the Shandong Analysis and Test Center.

### 3.2. Apparatus

TBE-300C HSCCC equipment was used with three multilayer columns of a total of 300 mL (the diameter of the PTFE tube was 2.6 mm) and a 20 mL sample loop ( Tauto Biotech, Shanghai, China). An LC3050N integrated system was used to contact the HSCCC equipment, composed with a P3050N gradient pump and a UV3050N monitor (Beijing Tong Heng Innovation Technology Co., Ltd., Beijing, China). A DC-0506 low-constant-temperature bath was used to control the temperature at 30 °C. An Agilent 1120 with a Zobax SB C_18_ column (5 μm, 250 mm × 4.6 mm, i.d.) was used for the sample detection process (Agilent Technologies, Santa Clara, CA, USA).

### 3.3. Preparation of Crude Extract

The crude sample of *T. asiatica* was prepared in our laboratory as previously described [22]. Firstly, the dried roots of *T. asiatica* (2 kg, 40~60 mesh) were extracted twice under reflux with 5 L of 95% ethanol (2 h each time). The extracts were filtrated, combined, and concentrated under vacuum by rotary evaporation at 50 °C. When the ethanol was removed, the pH was adjusted to 2 with 2% diluted hydrochloric acid. The solution was extracted three times with isometric petroleum ether, the pH of the degreased aqueous solution was slowly increased to 8.5 using aqueous ammonia, and the sample was extracted three times with isometric ethyl acetate. The upper ethyl acetate layer was combined and concentrated by rotary evaporation at 50 °C. Finally, the crude extract (6.3 g) was obtained after vacuum concentration and stored in a refrigerator at 5 °C.

### 3.4. Measurement of Partition Coefficient

The *K*_D_ values of the compounds were determined by HPLC. Firstly, different proportions of the solvent systems were prepared in a 5 mL tube and violently shaken. Two milliliters of each phase of the equilibrated two-phase solvent system was added to the crude extract (∼3 mg). After being shaken vigorously for 1 min and full separation achieved, 1 mL of each layer was removed and dried under nitrogen. The remnant was re-dissolved in 1 mL of methanol and analyzed by HPLC. The *K*_D_ value was defined as the peak area of a compound in the stationary phase divided by the peak area of a compound in the mobile phase with the following equation:

*K*_D_ = *A*_U_/*A*_L_

where *A*_U_ and *A*_L_ are the peak areas of target compounds in the stationary and mobile phases, respectively.

### 3.5. Preparation of Solvent System and Sample Solution

For the LGCCC elution, the solvent systems of *n*-hexane/ethyl acetate/methanol/water (5:5:1:9, *v*/*v*) and (5:5:4.5:5.5, *v*/*v*) were placed into the individed separating funnel. After being shaken vigorously, the solutions were kept still for several minutes and were separated into two phases for the experiment. The upper organic phase of *n*-hexane/ethyl acetate/methanol/water (5:5:1:9, *v*/*v*) was used as the stationary phase. The lower aqueous phase of *n*-hexane/ethyl acetate/methanol/water (5:5:1:9, *v*/*v*) was used as mobile A and (5:5:4.5:5.5, *v*/*v*) as mobile B. For the off-line 2D isocratic HSCCC elution, the solvent system of *n*-hexane/ethyl acetate/methanol/water (5:5:4:6, *v*/*v*) was placed into the separating funnel. After being shaken vigorously, the solutions were kept still for several minutes and were separated into two phases for the experiment. The upper organic phase was used as the stationary phase, while the lower aqueous phase was used as the mobile phase. All the stationary and mobile phases were ultrasonic degassed prior to use.

For the linear gradient HSCCC separation, 200 mg of the crude sample was dissolved in 10 mL of solution (1:1, upper:lower of HEMW at 5:5:1:9, *v*/*v*). For the off-line dimensional HSCCC separation, 45 mg of fraction A in the linear gradient eluton was set in 10 mL of isometric solution (1:1, upper:lower of HEMW at 5:5:4:6, *v*/*v*).

### 3.6. HSCCC Separation Procedure

For the LGCCC separation, the stationary phase was first introduced into the HSCCC column at a flowrate of 20.0 mL/min. The HSCCC equipment was rotated clockwise at 800 rpm at 30 °C, while mobile phase A was pumped into the CCC column at a flowrate of 5.0 mL/min in head-to-tail mode. After the establishment of a hydrodynamic equilibrium, the sample solution was injected through the injection valve. The prescribed mobile phase gradient combinations of A and B (Section 3.5) were applied, and the eluent was continuously monitored with a portable recorder at 320 nm. For the off-line dimensional HSCCC separation, the mobile phase with *n*-hexane/ethyl acetate/methanol/water (5:5:4:6, *v*/*v*) was set at a flowrate of 2.0 mL/min in head-to-tail mode and monitored with a portable recorder at 320 nm. All the fractions were collected in 10 mL test tubes according to UV absorption. The retention of the stationary phase was defined as the stationary phase relative to the total column capacity after separation.

### 3.7. HPLC Analysis

HPLC analyses of crude extract and CCC fractions were performed on an Agilent 1120 apparatus with a Zobax SB C_18_ column (5 μm, 4.6 mm × 250 mm, i.d.). The HPLC mobile phase comprised of water (A) and acetonitrile (B) with a linear gradient elution of 0–30 min, 22–55% B; 30–31 min, 55–95% B; and 34–35 min, 95–22% B. The flowrate was 1.0 mL/min, the selected wavelength was 320 nm, and the sample injection volume was 10 µL.

### 3.8. Structural Identification

The separated compounds were identified by ESI-MS as well as ^1^H and ^13^C NMR spectra. The ESI-MS experiment was performed on an Agilent 6520 Q-TOF (Agilent, Santa Clara, CA, USA). NMR spectra were recorded on a Bruker AV-400 spectrometer (Bruker BioSpin, Rheinstetten, Germany) with DMSO. Solvent and chemical shift (*δ*) values are expressed, respectively, in parts per million (ppm) and coupling constant (*J*) in Hz.

## 4. Conclusions

The current study demonstrates an alternate and more efficient separation method using two-dimensional HSCCC linear gradient and isocratic elution modes for the separation of the main components from *T. asiatica*. Similar to a binary gradient in HPLC, the solvent system was varied by adjusting the proportions of the two solvent systems (lower phase of *n*-hexane/ethyl acetate/methanol/water 5:5:1:9 and 5:5:4.5:5.5). Furthermore, a mixture was separated by off-line 2D-CCC. Six main coumarin moieties, namely, tomentin (**1**), toddalolactone (**2**), 5,7,8-trimethoxycoumarin (**3**), mexoticin (**4**), isopimpinellin (**5**), and toddanone (**6**), were efficiently separated, with purities above 98%. The established LG and 2D-CCC provided an efficient method for separating coumarins from *T. asiatica*. It may also be applied to separate compounds with broad *K*_D_ values from other complex natural products.

## Figures and Tables

**Table 1:** The *K*_D_ values of target compounds in HSCCC with different solvent systems.

Compound No.	*K*_D_ Values (*n*-Hex/EtOAc/MeOH/H_2_O, *v*/*v*)						
	5:5:1:9	5:5:2:8	5:5:3:7	5:5:4:6	5:5:4.5:5.5	5:5:5:5	5:5:6:4
**1**	1.74	0.90	0.66	0.33	0.24	<0.20	<0.20
**2**	0.94	0.53	0.40	0.21	<0.20	<0.20	<0.20
**3**	>5.00	4.27	2.94	1.17	0.94	0.54	0.25
**4**	>5.00	>5.00	3.23	1.28	1.05	0.62	0.30
**5**	>5.00	>5.00	>5.00	2.89	2.35	1.25	0.52
**6**	>5.00	>5.00	>5.00	3.20	2.68	1.49	0.68

## Data Availability

The data presented in this study are available in article.

## Sample Availability

Samples of the compounds are available from the authors.
